# Impact of Preweaning Vaccination on Host Gene Expression Patterns Linked to Future Bovine Respiratory Disease Development in Beef Calves

**DOI:** 10.3390/vaccines14080694

**Published:** 2026-08-12

**Authors:** Hudson R. McAllister, Bradly I. Ramirez, Sarah F. Capik, Kelsey M. Harvey, Paul S. Morley, Robert J. Valeris-Chacin, Brandi B. Karisch, Amelia R. Woolums, Alexis C. Thompson, Matthew A. Scott

**Affiliations:** 1 Veterinary Education, Research, and Outreach Program, Texas A&M University, Canyon, TX 79015, USA; 2 Tumbleweed Veterinary Services PLLC, Amarillo, TX 79118, USA; 3 Department of Veterinary Pathobiology, College of Veterinary Medicine, Oklahoma State University, Stillwater, OK 74078, USA; 4 Prairie Research Unit, Mississippi State University, Prairie, MS 39756, USA; 5 Department of Animal and Dairy Sciences, Mississippi State University, Starkville, MS 39762, USA; 6 Department of Pathobiology and Population Medicine, Mississippi State University, Starkville, MS 39762, USA; 7 Texas A&M Veterinary Medical Diagnostic Laboratory, Canyon, TX 79016, USA

**Keywords:** bovine respiratory disease, transcriptomics, beef cattle, preweaning vaccination, inflammation, RNA sequencing, gene expression, immune development

## Abstract

Background/Objectives: Bovine respiratory disease (BRD) remains a major concern in cattle research, and the long-term effects of vaccination on health and immune responses are not well defined. This study compared gene expression in vaccinated (VAX) and unvaccinated (NOVAX) preweaned calves and subsequent BRD development during backgrounding. Methods: Whole blood was collected at four timepoints (TIME; T1-4; median age 107, 114, 183, and 230, respectively) from 73 bull calves enrolled in a blinded randomized controlled trial; VAX calves received a commercial attenuated multivalent viral vaccine at T1 and T3. Results: Whole-blood transcriptomics was used to quantify mRNA, identifying 5364 differentially expressed genes (DEGs) for TIME, 84 DEGs for vaccination (VAX), and 129 for BRD status using both glmmSeq and QLF testing (glmmSeq only DEGs: 11,068 TIME, 358 VAX, and 9241 BRD). VAX calves at T3 were clustered uniquely with the enrichment of pathways related to the cellular response to stress, neutrophil degranulation, and antigen processing and presentation compared to NOVAX cattle and VAX at other timepoints. Interferon pathways, natural killer cell responses, and neutrophil activity were generally absent across all timepoints, while antigen presentation pathways were persistently enriched. Regardless of vaccination or future BRD diagnosis, immunological development over time was indicated by DEGs related to adaptive immunity, lymphocyte development, and inflammatory resolution. At T4, cattle diagnosed with BRD during backgrounding had differential gene expression related to oxygen transport, hemoglobin function, and metabolic processes compared to cattle that remained healthy. Conclusions: This study provides insights into the possible genomic mechanisms underlying vaccine responses and preclinical BRD susceptibility in preweaned beef cattle.

## 1. Introduction

Bovine respiratory disease (BRD) remains the leading cause of morbidity and mortality in North American beef cattle production systems, accounting for approximately 70–80% of feedlot mortality and over $1 billion in annual economic losses [1,2]. The disease complex involves interactions between viral pathogens, bacterial pathogens, environmental factors, and host immune responses [3,4]. Peak BRD incidence often occurs during background and feedlot entry periods, when cattle experience concurrent stressors, including weaning, transport, commingling, and abrupt dietary changes [4]. Current BRD prevention strategies rely heavily on vaccination programs, including viral and bacterial vaccines, and metaphylactic antimicrobial administration [5,6]. While vaccines aid in reducing BRD incidence, substantial variation in individual vaccine response and disease susceptibility is evident [7,8]. This heterogeneity likely reflects complex interactions between host genetics, developmental immune status, environmental factors, and pathogen exposure intensity [8]. Increasing regulatory and consumer pressure to reduce antimicrobial use in livestock production has intensified the need for an improved understanding of alternative strategies for identifying and managing cattle that are at high risk for BRD.

Whole-blood transcriptomics offers a powerful approach to understanding the host immune response and identifying molecular biomarkers of vaccine responsiveness and disease susceptibility. Previous studies have demonstrated that gene expression signatures can predict vaccine-induced antibody responses in humans and distinguish disease states in cattle [9,10,11,12]. However, most bovine transcriptome studies have employed cross-sectional designs or limited sampling timepoints, excluding the comprehensive characterization of developmental immune patterns and their interactions with vaccination and disease outcomes [12]. Critical knowledge gaps in the current literature remain regarding (1) how peripheral blood immune transcriptomes evolve during the neonatal to juvenile transition in beef cattle; (2) whether vaccination-induced transcriptional signatures vary across developmental stages; (3) whether preclinical transcriptomic biomarkers can identify animals at elevated BRD risk before the onset of clinical disease; and (4) whether vaccination status modifies disease-associated molecular patterns. Addressing these gaps requires longitudinal sampling across developmental phases, vaccination events, and disease outcomes within individual animals [13].

Building on our previous pilot work characterizing preweaning vaccination effects on host gene expression [14], the present study extends this analysis to include more animals to increase the statistical power of our findings and includes calves that would eventually become diseased, seeking to better understand the host transcriptome and disease outcome relationship. The objective of this study was to characterize peripheral blood transcriptomes longitudinally from birth through weaning in beef cattle, examining the interactive effects of attenuated vaccination status and subsequent BRD diagnosis during backgrounding. Using whole-blood transcriptomics, we sampled 81 calves at four timepoints from birth to weaning, recording BRD outcomes during the subsequent backgrounding phase following weaning. This structure allowed us to address two objectives: (1) to determine the influence of vaccination and marketing strategies on host gene expression through whole-blood transcriptomics and (2) to determine whether preweaning peripheral blood gene expression was associated with BRD diagnosis during the subsequent backgrounding phase. We hypothesized that (1) the administration of a commercially available vaccine against BRD-associated pathogens and exposure to commercial marketing systems as opposed to direct purchasing would induce a distinct host transcriptome signature and (2) transcriptomic profiles measured before weaning would differ between calves that were subsequently diagnosed with BRD and those that were not. This work continues and expands comprehensive longitudinal blood transcriptome work spanning the birth to weaning period in beef cattle, characterizing patterns of developmental immunology and providing a hypothesis-generating basis for future research in precision livestock health management.

## 2. Materials and Methods

### 2.1. Animal Use and Previous Work

All animal use and procedures were approved by the Mississippi State University and West Texas A&M University Animal Care and Use Committee (IACUC protocols #19-169 and #2019.04.002, respectively) and were carried out according to relevant IACUC and agency guidelines. In addition, this study is reported in accordance with the Animal Research: Reporting of In Vivo Experiments (ARRIVE) guidelines [15]. This cohort has been described in previous work examining BRD morbidity, mortality, and performance [16]; an initial transcriptomic comparison of vaccinated and unvaccinated calves among healthy calves [14]; and the interaction of marketing strategies and vaccination on the backgrounding-entry transcriptomes [17]. The present study is distinct in scope because it characterizes whole-blood transcriptomic changes across the preweaning period (T1–T4, birth through weaning) in the full cohort, including calves that subsequently developed BRD during the backgrounding phase; the marketing strategy and the postweaning backgrounding phase are not evaluated here.

### 2.2. Treatment Allocation

Eighty-one commercial cross-bred bull calves were randomly assigned in a split-plot randomized controlled trial to evaluate gene expression pathways in vaccinated and unvaccinated calves that remained healthy or later developed BRD. At the conclusion of the calving season, bull calves were assigned to the whole-plot assignment to receive a subcutaneous attenuated viral respiratory vaccine and booster (2 mL SQ; Pyramid 5, Boehringer Ingelheim Animal Health, Duluth, GA, USA) or a subcutaneous sterile saline injection (0.9% saline solution) (VAX; *n* = 39 or NOVAX; *n* = 42, respectively). Throughout the cow–calf production phase, calves were housed in six grass-lot pastures (*n* = 14 cow–calf pairs per pasture), grouped by VAX (*n* = 3 pastures) or NOVAX (*n* = 3 pastures), for approximately 230 days. Additional cow–calf pairs were housed in the same pens but were not enrolled in the study (*n* = 6 to 9 pairs). Each additional cow–calf pair underwent the same vaccination protocol as the study animals housed in the same pasture.

### 2.3. Marketing Strategy, Sampling Timepoints, and BRD Case Definition

At the end of the cow–calf phase, steer calves were then assigned to the split-plot-level treatment, where they were either weaned and housed at the Mississippi Agricultural and Forestry Experiment Station Prairie Research Unit (Prairie, MS, USA) for three days before direct shipment to Texas (Texas A&M AgriLife Bushland Research Feedlot, Bushland, TX, USA; DIRECT *n* = 42) or transported to a commercial auction facility in North Mississippi, housed in pens for approximately 6 h, transported to a regional order-buyer facility for three days, and then shipped to Texas (AUCTION *n* = 39). All researchers and staff responsible for assessing the health of cattle were blinded to the treatment groups (vaccination and marketing strategy) during data collection. Calves were sampled at four timepoints: T1, vaccination (median age = 107 days); T2, seven days postvaccination (median age = 114 days); T3, revaccination and surgical castration (median age = 183 days); T4, weaning (median age = 230 days) (Supplemental File S1). These four timepoints were selected because each corresponds to a routine, industry-relevant management event at which calves in commercial cow–calf systems are gathered and restrained: initial vaccination (T1), seven days after initial vaccination with the goal of capturing transcriptional changes from the vaccine (T2), revaccination and surgical castration (T3), and weaning immediately before entry into the backgrounding phase. Sampling at these timepoints was intended to ensure that any candidate biomarker identified could plausibly be collected under commercial conditions without imposing additional handling events.

Jugular blood samples were taken at each timepoint prior to treatment and management tactics into Tempus Blood RNA tubes (Applied Biosystems, Waltham, MA, USA). At T1, calves were tested via ear-notch ELISA for bovine viral diarrhea virus (BVDV) persistent infection and received a multivalent respiratory vaccine according to their assigned treatment group, and all calves received a multivalent clostridial bacterin–toxoid subcutaneously; there were no BVDV-positive calves. At T3, calves were revaccinated identically to T1 with the treatment vaccine and bacterin–toxoid and surgically castrated. Groups (VAX and NOVAX) were maintained without direct contact during sampling and in pastures. In the 45-day backgrounding phase following T4, cattle were examined daily by trained caretakers for signs of BRD and treated based on a standard protocol [18]. Cattle were classified as BRD (treated for BRD during backgrounding, *n* = 32) or NO BRD (not treated for BRD during backgrounding, *n* = 49). Importantly, no calf was diagnosed or treated for clinical BRD during the cow–calf phase (T1–T4).

### 2.4. Next-Generation RNA Sequencing and Bioinformatic Data Processing

Using the Tempus Spin RNA Isolation Kit (Applied Biosystems, Waltham, MA, USA), total RNA was isolated according to the manufacturer’s instructions. Each sample’s total RNA was analyzed for RNA concentration and integrity using a Qubit 4.0 Fluorometer (ThermoFisher, Waltham, MA, USA) and an Agilent 4400 Bioanalyzer (Agilent Technologies, Santa Clara, CA, USA). All RNA samples were of high quality (RIN mean = 8.92, s.d. = 0.36) and concentration (ng/μL mean = 182.30, s.d. = 111.79). Library preparation and RNA sequencing were performed by Texas A&M Institute for Genome Sciences and Society (TIGSS; College Station, TX, USA). Following the manufacturer’s instructions, library preparation for mRNA was performed with the Stranded mRNA Library Prep Kit (Illumina, San Diego, CA, USA). Prepared libraries were sequenced with an Illumina NovaSeq 6000 analyzer (v.1.7+, S4 reagent v1.5; San Diego, CA, USA) in six flow cell lanes, utilizing paired-end sequencing for 150 base pair read fragments. Of the 81 enrolled calves, 73 animals were selected for RNA sequencing. All 32 calves treated for BRD during the subsequent backgrounding phase were included with 41 of the 49 calves that were never treated, selected with approximately equal representation of the vaccination groups (VAX, 33 of 39; NOVAX, 40 of 42); the remaining eight untreated calves were not sequenced. A total of 292 samples were sequenced and included in the analysis, representing 73 animals across 4 timepoints (NOVAX; *n* = 40; VAX; *n* = 33); seven samples failed to cluster and were subsequently removed from downstream analyses (J013 at T2 (NOVAX DIRECT), J018 at T3 (NOVAX DIRECT), J019 at T3 (NOVAX DIRECT), J025 at T3 (VAX AUCTION), J059 at T3 (NOVAX AUCTION), J117 at T3 (NOVAX AUCTION), and J122 at T1 (VAX DIRECT)).

Read quality was assessed with FastQC v0.11.9 and MultiQC v1.12, and read pair trimming for ambiguous bases and adaptor sequences, retaining a minimum length of 28 bases, was performed using Trimmomatic v0.39 [19,20,21]. Trimmed reads were processed through ARS-UCD2.0 reference-guided assembly with HISAT2 v2.2.1 [22]. Sequencing yielded a mean of approximately 34 million paired-end reads per sample (range: 22 million–56 million), and a mean overall alignment rate of 96% to the ARS-UCD2.0 reference (range: 93–98%). Using Samtools v1.14, sequence alignment/map (SAM) files were converted to binary alignment map (BAM) files [23]. Transcript assembly and gene-level expression estimation for differential expression analysis were performed using StringTie v2.1.7 [24]. Previous raw sequencing data that were incorporated into this work are discussed elsewhere and can be found at the National Center for Biotechnology Information Gene Expression Omnibus (NCBI-GEO) under the accession number GSE205004 [14]. The new raw RNA sequencing data produced in this study are publicly available at the NCBI-GEO under the accession number GSE248477 [25].

### 2.5. Differential Gene Expression Analysis

Gene-level count matrices were processed and analyzed in RStudio, using R v.4.2.1. Following sample classification, the ComBat_seq function in the package sva v3.52.0 was utilized for batch effect adjustment, in the form of sequencing runs (data from GSE205004 as “1” and new data generated under GSE248477 as “2” (“Batch”; Supplemental File S2)), using an empirical Bayes framework for the raw gene counts [26,27]; this was applied to all sequencing libraries simultaneously. Raw gene counts were processed and filtered via the procedures described by Chen et al., following classification by vaccination group and timepoint. Samples were retained if they had a total count greater than 100 and a count per million (CPM) of at least 0.2 in at least twelve samples [26]. Following filtering, the dataset was considered non-sparse and normalized for differential expression analysis with the trimmed mean of M-values (TMM) [27]. Tagwise dispersion estimates of gene counts were supplied to the Bioconductor package glmmSeq v0.1.0 for the negative binomial mixed-effect modeling of gene counts. A single model was fitted for all genes, with timepoint (TIME), vaccination (VAX), and subsequent BRD treatment (BRD) as fixed effects, together with their two-way interactions, and pasture and animal ID as random intercepts. All fixed effects and interactions were estimated simultaneously rather than in separate models. Pasture assignment was fixed for the duration of the preweaning phase, animal was nested within pasture by design, and the animal-level random intercept accounted for the repeated-measures correlation among the four samples from each calf.

Following model adaptation, differentially expressed genes (DEGs) were assessed across timepoints, vaccine groups, BRD treatment, and the interactions between vaccine, timepoint, and BRD treatment, with *p*-values adjusted for false discovery rates (FDRs) using the Benjamini–Hochberg method. Genes were considered significantly expressed with an FDR ≤ 0.05. Pairwise comparisons for DEGs between each vaccination group at each timepoint were performed using edgeR v3.36.0, fitting genes under a generalized linear framework and employing quasi-likelihood F-tests (QLF); pairwise interactions were considered significant with an FDR ≤ 0.10 [28]. The more permissive a priori threshold was applied to the pairwise QLF tests because these comparisons were performed within a single timepoint and had fewer degrees of freedom than in the mixed model fitted across all four timepoints; the reasoning for the pairwise step was to retain candidate genes for the subsequent intersection rather than to declare them significant in isolation. Together, these analyses evaluated differential expression in two complementary ways: (1) a generalized linear mixed model (glmmSeq) testing for the overall effects of time, vaccination, and BRD status while accounting for random effects and (2) pairwise edgeR-compared treatment groups at individual timepoints. Genes were carried forward into functional enrichment analysis only when identified by both frameworks. This intersection was applied deliberately as a conservative filter rather than as a formal significance criterion, and it accounted for the difference between the number of genes identified by glmmSeq alone and the number retained after intersection. Genes identified by only one method can be found in Supplemental Files S3–S5 and were not solely interpreted. Reported DEG counts should be read as a conservative approach. BRD incidence and treatment occurred later, during the postweaning–backgrounding phase, meaning that BRD status is a retrospectively assigned label describing a subsequent clinical outcome, rather than a condition present at the time of sampling. BRD was included as a fixed effect so that gene expression measured before disease could be compared between animals that did or did not subsequently receive treatment for BRD. Accordingly, BRD DEGs describe associations with a later outcome and are not predictive of it.

A priori sample size and power calculations were performed at the design stage using RnaSeqSampleSize [29] and parameterized with pilot RNA-seq data from this cohort [14]. Assuming a minimum average read count of 200 among prognostic genes, a maximum dispersion between samples of 0.4, the ratio of the geometric mean of normalization factors of 1.2, a minimum between-sample fold change of 2, and an FDR of 0.05, approximately 36 animals per comparison group would yield 98% statistical power to detect differential gene expression. The contrasts analyzed here approximate but do not exactly match the balanced design: the BRD contrast comprised 32 vs. 49 calves and the VAX contrast comprised 32 vs. 40 calves. The achieved power for the unbalanced contrasts may be considered lower than the design-stage estimate and lower again for the BRD × VAX interaction, which subdivided the cohort further.

### 2.6. Dimensional Reduction and Unsupervised Clustering Analyses

Filtered and log2 count-per-million (log2CPM) values of TMM-normalized gene counts from all samples were used to conduct heatmap, principal component, and clustering analyses. The Bioconductor package pheatmap v.1.0.12 was used to create a heatmap and perform the exploratory clustering of samples with reference to vaccination, timepoints, and individual animal IDs, using Canberra distances for the unsupervised hierarchical clustering of samples and Pearson correlation coefficients for DEGs. Z-scores were calculated and used for heatmap analysis from the log2CPM values of TMM-normalized expression values. Gene expression was grouped into 36 distinct clusters using the k-means algorithm embedded within pheatmap; cluster numbers were determined using the Elbow method. Principal component analysis for high-dimensional data exploration and reduction was conducted using the Bioconductor package PCAtools v2.0.0 with a correlation matrix; normalized gene counts were processed through mean centering and variance scaling. A scree plot was generated to evaluate the number of principal components to retain for analysis using the Elbow and Horn’s parallel analysis methods [30]. A Spearman’s rank correlation matrix of retained PCs was constructed using the metadata components vaccination group (VAX) and BRD acquisition during the backgrounding phase (BRD) from each timepoint; correlations were considered significant with an FDR ≤ 0.10. Following PC retainment, a PCA biplot was created for each timepoint to evaluate sample dissimilarities based on BRD and VAX status; data ellipses were calculated from multivariate t-distributions across each timepoint, encompassing 80% confidence levels of the expressional t-distributions.

### 2.7. Functional Enrichment Analyses of DEGs

Functional enrichment for gene ontology (GO), Reactome pathways, and KEGG pathways of DEGs were analyzed using KOBAS-i via hypergeometric testing and Benjamini–Hochberg-adjusted *p*-values (FDR ≤ 0.05) [31]. Functional enrichment of DEGs was analyzed via (1) DEGs shared between timepoints in both glmmSeq and QLF testing of VAX and NOVAX calves to identify shared genes between timepoints in both QLF and glmmSeq analyses; (2) DEGs between treatment groups VAX vs. NOVAX across each timepoint by both glmmSeq and QLF testing, removing DEGs identified in method 1; (3) DEGs identified only in glmmSeq for timepoint–VAX interactions, allowing for an independent evaluation of functional enrichment influenced by calf development and vaccine administration. Using enriched GO terms, directionality was evaluated based on the log2 fold changes of associated DEGs.

## 3. Results

### 3.1. Differential Gene Expression Overview

The analysis of genes from glmmSeq resulted in 11,068, 358, and 9241 for TIME, VAX, and BRD, respectively. Between glmmSeq and QLF testing, a total of 5364, 84, and 129 DEGs were identified for TIME, VAX, and BRD, as shown in Supplemental Files S3–S5. The interaction of BRD × TIME resulted in 307 DEGs, and TIME × VAX resulted in 3001 DEGs, as shown in Supplemental File S6. All functional enrichments of DEGs in the same categories of TIME, BRD, and VAX are reported in Supplemental Files S7–S9, respectively. Representative DEGs for each major comparison, with their direction of regulation and biological relevance, are summarized in Table 1.

### 3.2. Time-Dependent Gene Expression Patterns

**Figure 1:**
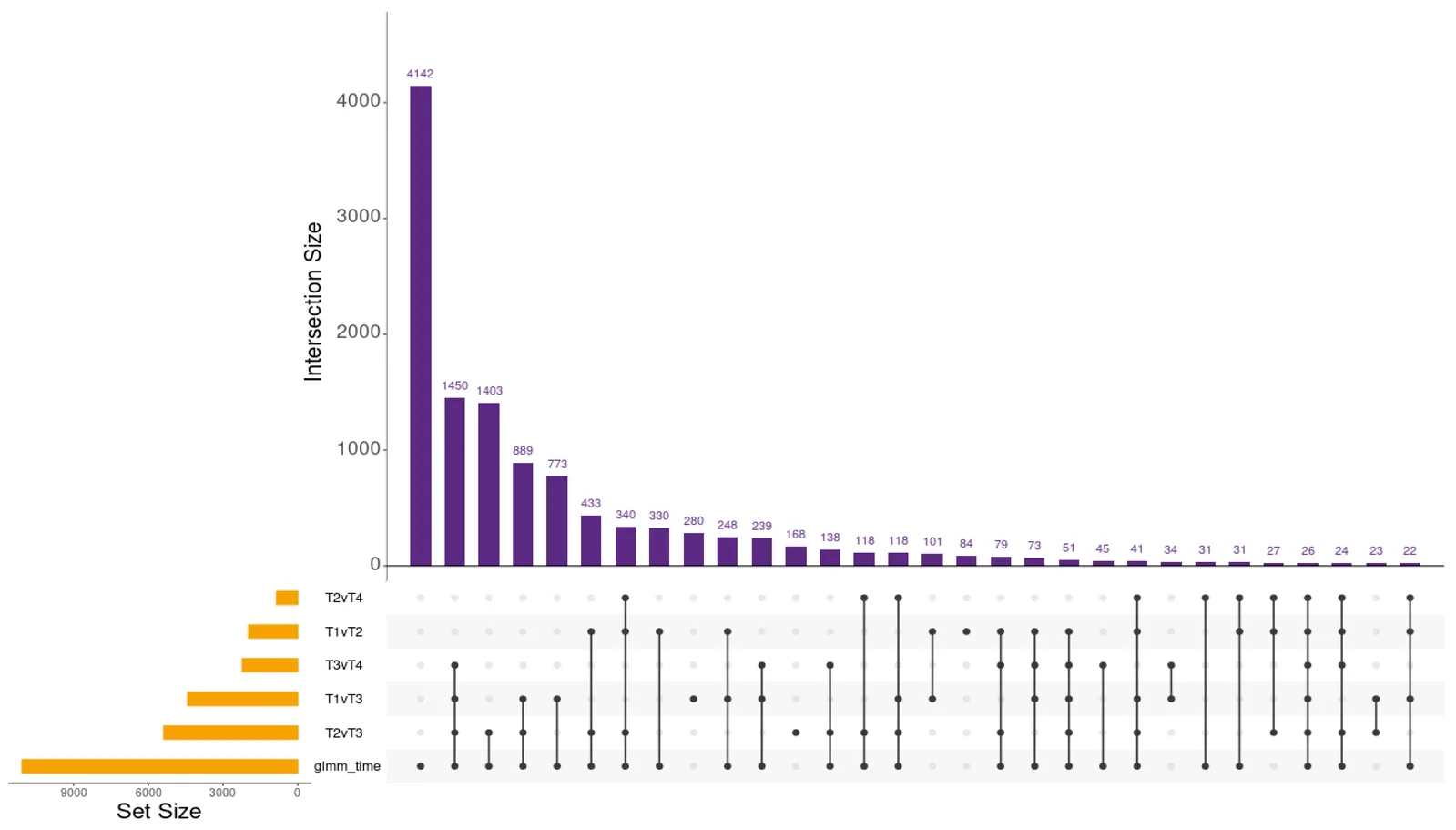
UpSet plot and differential gene expression summary across pairwise timepoint comparisons and the overall TIME effect from the generalized linear mixed model (glmm_time). The T1vT4 pairwise comparison (*n* = 14 DEGs) was excluded from visualization due to negligible size but is included in the reported union total. Horizontal bars on the left represent the total number of genes identified in each individual set. Vertical bars at the top of the matrix indicate the size of each intersection between selected sets, with connected dots below each bar showing which sets are involved in that intersection. In immunological terms, the glmm time set represents all genes whose expression changed significantly across the preweaning period after accounting for vaccination and BRD status, whereas each pairwise set represents genes that differed between two specific timepoints.

Time was a major driver of gene expression variation across all treatment groups. Across all timepoints, 11,068 DEGs were expressed when accounting for vaccination and BRD status (Figure 1, Supplemental File S3). Key enriched pathways included immune system, innate immune system, adaptive immune system, and neutrophil degranulation (Supplemental File S7). Cytokine signaling and interleukin signaling were also upregulated over time. Additionally, biosynthesis of specialized pro-resolving mediators was upregulated, including genes such as ALOX5 and ALOX15, as well as pathways involved in the synthesis of leukotrienes and eoxins, arachidonic acid metabolism, and the biosynthesis of D-series resolvins and protectins (Table 1). Key GO terms included cytoplasm (GO:0005737), protein binding (GO:0005515), positive regulation of transcription by RNA polymerase II (GO:0045944), B-cell receptor signaling pathway (GO:0050853), and platelet activation (GO:0030168). Gene expression related to these pathways was upregulated from T1 to T3 and then downregulated to T4 (Supplemental File S7). This pattern was observed in pathways related to neutrophil degranulation, cytokine signaling, adaptive immune responses, and specialized pro-resolving mediator biosynthesis, suggesting an elevation in immune system activation at 183 days of age (T3), sampled before that day’s revaccination and castration, followed by the resolution of inflammation signaling by weaning at T4 (230 days). A further finding was the enrichment of specialized pro-resolving mediator (SPM) biosynthesis pathways, including resolvin, protectin, and arachidonic acid metabolism. Sampling at T3 preceded the attenuated viral booster and surgical castration performed that followed.

**Figure 2:**
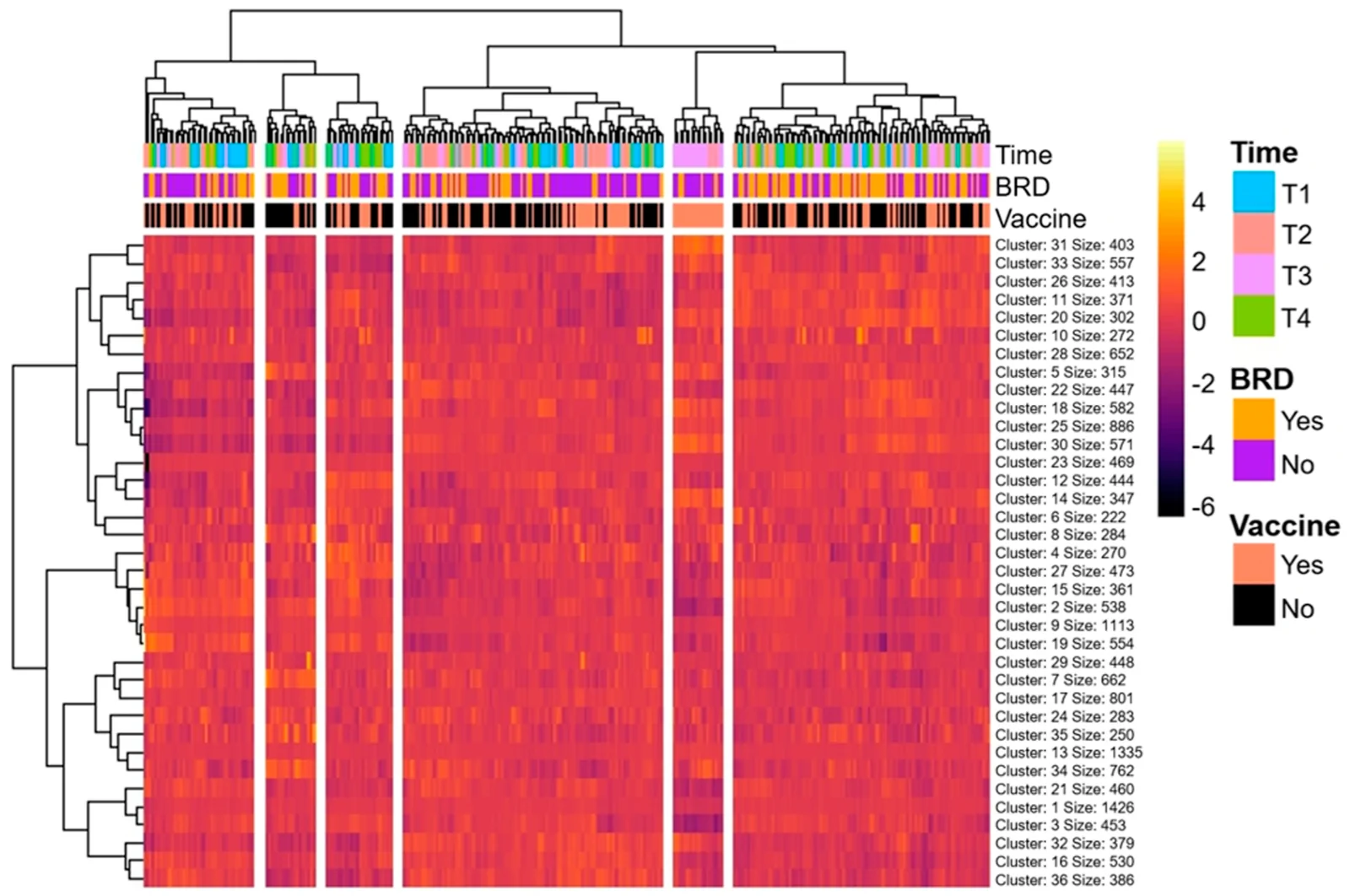
Hierarchical clustering heatmap of gene expression across timepoints, BRD status, and vaccine status in preweaning beef calves. Gene clusters (rows) and samples (columns) were hierarchically clustered based on z-score-normalized gene expression values using the Canberra distance. Color annotations above the heatmap denote metadata categories: timepoint (T1 (median age = 107 days): turquoise; T2 (median age = 114 days): salmon; T3 (median age = 183 days): lavender; T4 (median age = 230 days: green)), bovine respiratory disease (BRD) status (yes: orange; no: purple), and vaccine status (vaccinated [VAX]: pink; unvaccinated [NOVAX]: black). Expression intensity is color-coded using a diverging scale (yellow/orange = high expression; dark blue/black = low expression) based on z-score-normalized values. Gene clusters are annotated with size labels indicating the number of genes per k-means cluster (*n* = 36).

A set of 5364 TIME DEGs was identified as the largest source of differential expression by both GLMM and at least one pairwise comparison. Hierarchical clustering analysis using the Canberra distance metric demonstrated the distinct separation of samples by timepoint, with T3 samples showing the greatest divergence from all other samples (Figure 2). Using principal component analysis, timepoint was a primary driver of variation within the dataset. The separation of clusters were assessed visually; no formal statistical test of cluster separation was performed, and the clustering and ordination results presented throughout are descriptive rather than inferential.

### 3.3. Vaccination-Associated Gene Expression

**Figure 3:**
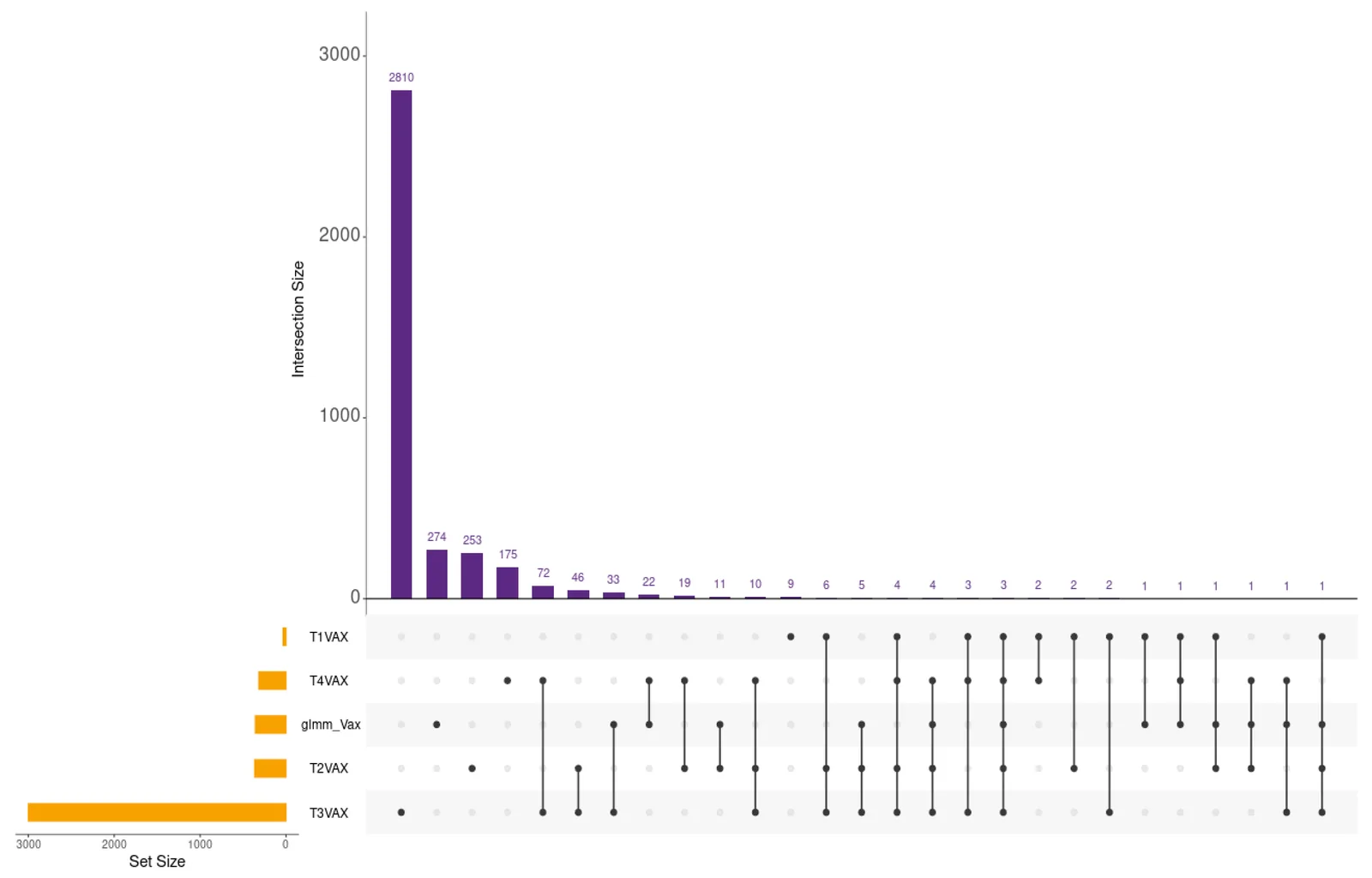
UpSet plot and differential gene expression summary across pairwise timepoint comparisons and the overall VAX effect from the generalized linear mixed model (glmm_Vax). Horizontal bars on the left represent the total number of genes identified in each individual set. Vertical bars at the top of the matrix indicate the size of each intersection between selected sets, with connected dots below each bar showing which sets are involved in that intersection. In immunological terms, the glmm_Vax set represents all genes whose expression changed significantly across the preweaning period after accounting for TIME and BRD status, whereas each pairwise set represents genes that differed between two specific timepoints.

**Figure 4:**
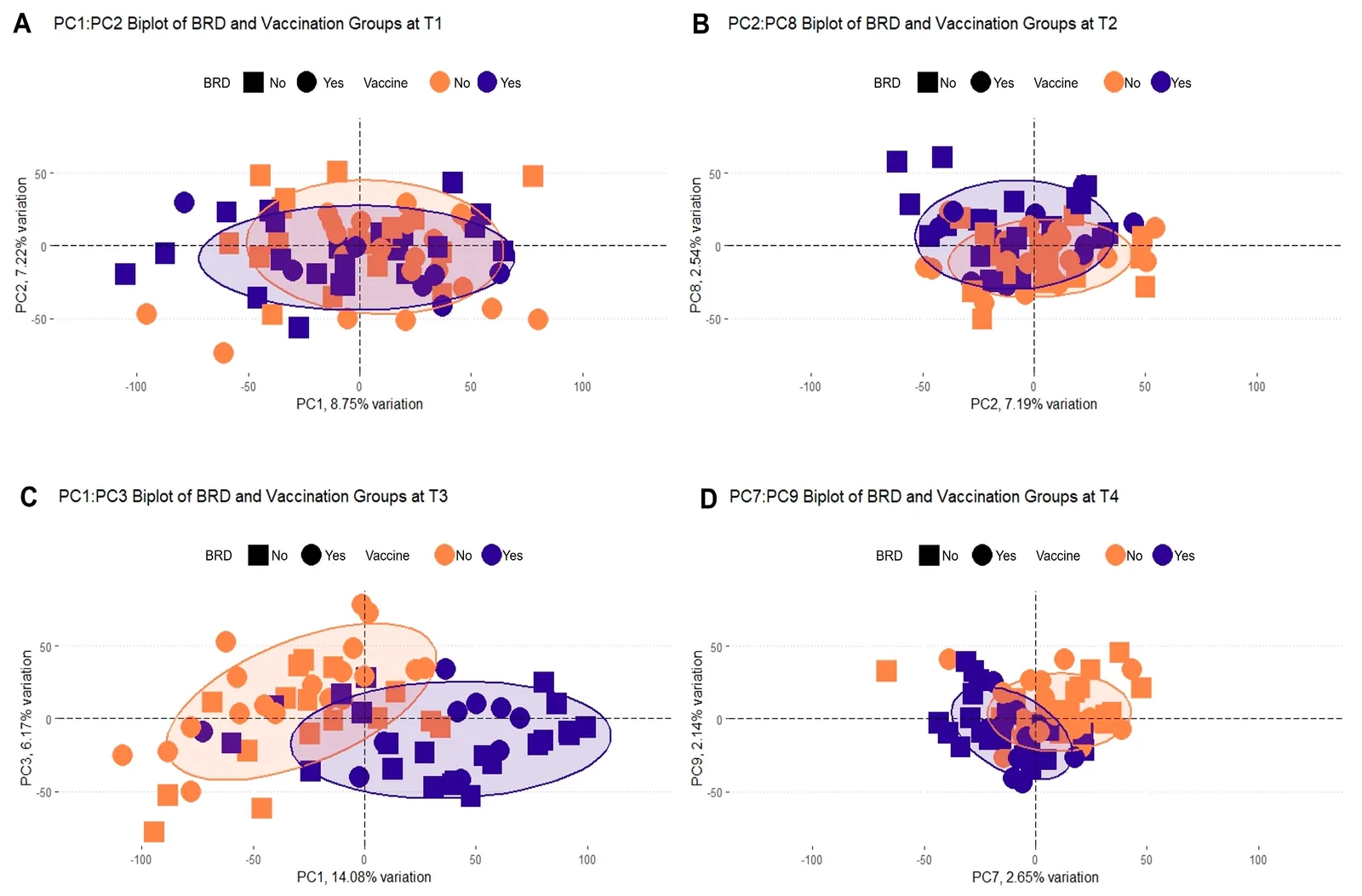
Principal component analysis (PCA) biplots of principal components (PCs) significantly associated with bovine respiratory disease (BRD) status and/or vaccination status at (**A**) T1 (107 days of age), (**B**) T2 (approximately 114 days), (**C**) T3 (approximately 183 days), and (**D**) T4 (approximately 230 days, weaning). PC pairs shown at each timepoint were selected based on significant associations with BRD and/or vaccine status. Individual sample points are colored by vaccination status (purple = vaccinated [VAX]; orange = unvaccinated [NOVAX]) and shaped by BRD outcome (square = clinically healthy [NOBRD]; circle = BRD-diagnosed [BRD]). Confidence ellipses (80%) are shown for each group. Axis labels indicate the percentage of total variance explained by each PC.

A total of 84 DEGs identified by both models were enriched related to vaccination across all timepoints, when accounting for TIME and BRD (Figure 3, Supplemental File S5). At T3, before revaccination at approximately 183 days of age, VAX cattle had increased gene expression pathways compared to NOVAX cattle. Enriched pathways at T3 included cellular stress response, such as the regulation of the HSF1-mediated heat shock response (*HSPA4*), neutrophil degranulation, antigen processing and presentation, and Fc-gamma receptor-dependent phagocytosis (Table 1; Supplemental File S9). Global gene expression patterns indicated that VAX cattle at T3 were clustered together relative to all other samples (Figure 2). Unsupervised hierarchical clustering revealed T3 VAX samples clustering from all other timepoints of both the VAX and NOVAX groups and T3 NOVAX samples. Principal component analysis was consistent with a distinct profile for T3 VAX cattle (Figure 4C). In contrast, VAX and NOVAX cattle showed overlapping distributions at T1 (Figure 4A), T2 (Figure 4B), and T4 (Figure 4D), indicating that the most pronounced vaccine-associated transcriptional differences measured in this study occurred approximately two months after initial vaccination, immediately prior to revaccination.

Enrichment related to classical antiviral pathways was not detected at any timepoint. Type I interferon pathways, interferon-gamma response pathways, natural killer cell responses, and sustained neutrophil activity were not significantly enriched when comparing VAX to NOVAX cattle (Supplemental File S9). Antigen presentation pathways, by contrast, were persistently enriched across timepoints (Supplemental File S9).

### 3.4. BRD-Associated Gene Expression

**Figure 5:**
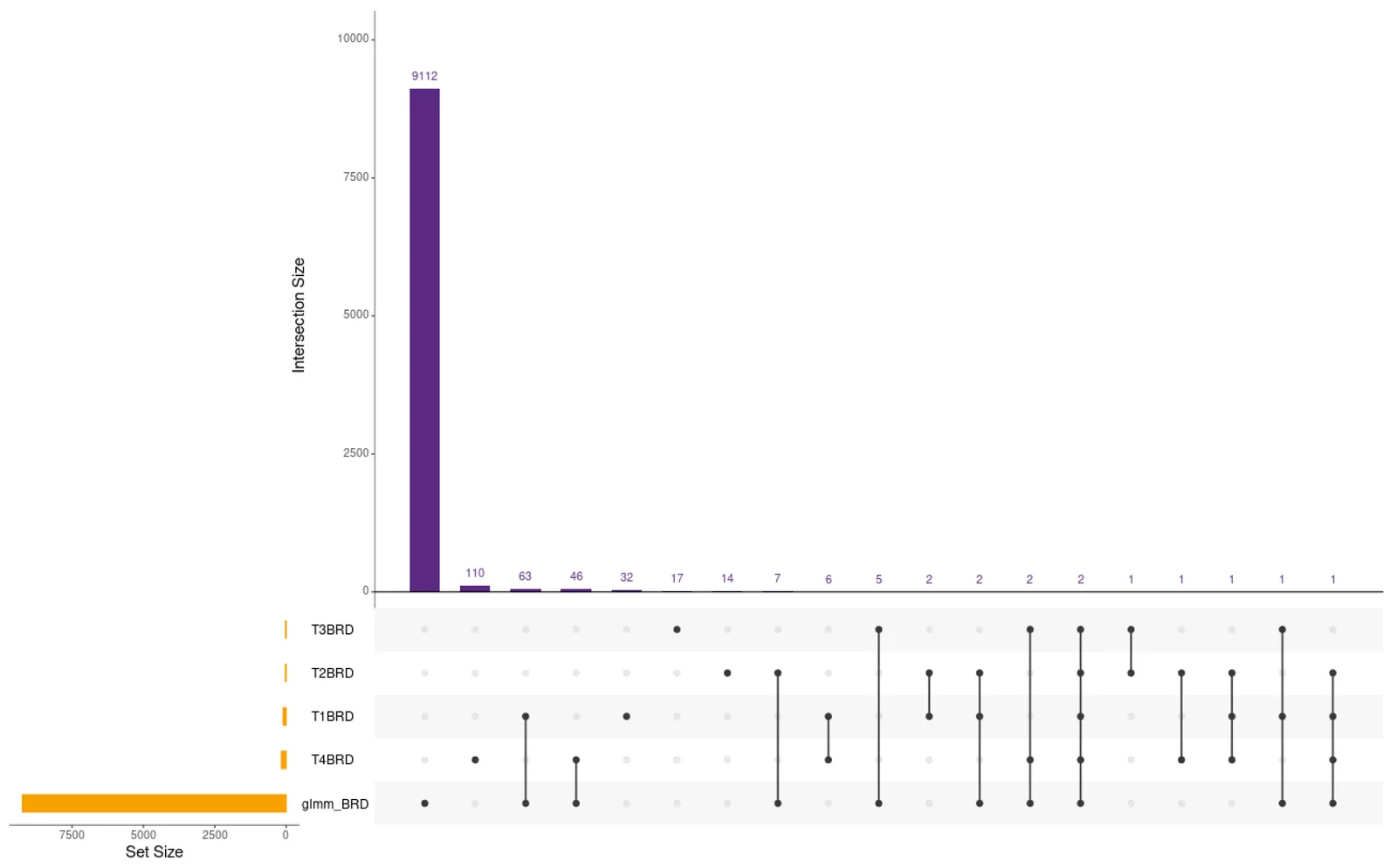
UpSet plot and differential gene expression summary across pairwise timepoint comparisons and the overall BRD effect from the generalized linear mixed model (glmm_BRD). Horizontal bars on the left represent the total number of genes identified in each individual set. Vertical bars at the top of the matrix indicate the size of each intersection between selected sets, with connected dots below each bar showing which sets are involved in that intersection. In immunological terms, the glmm_BRD set represents all genes whose expression changed significantly across the preweaning period after accounting for TIME and VAX status, whereas each pairwise set represents genes that differed between two specific timepoints.

A total of 129 DEGs were enriched by both analysis techniques across all timepoints for BRD when comparing cattle that remained healthy or developed BRD during the backgrounding phase (Figure 5, Supplemental File S5). At T4, when weaning occurred (230 days) and immediately before the backgrounding period, cattle that eventually were diagnosed with BRD (within 45 days of arrival at the backgrounding yard) demonstrated altered expression of genes compared to cattle that stayed healthy (i.e., were not treated for BRD) during backgrounding [13,14]. Gene expression pathways for BRD cattle at T4 were related to oxygen transport and hemoglobin function, such as hemopoiesis, hemoglobin complex and metabolic process, and O_2_ and CO_2_ exchanged in erythrocytes (Supplemental File S8). Influential genes were *RHAG*, *AHSP*, and *SPTA1* (Table 1).

Overall, cattle that ultimately were affected by BRD during backgrounding demonstrated altered expression of genes for pathways related to cellular metabolism, including fatty acid metabolism and oxidation–reduction processes, and diseases of metabolism, driven by genes such as *MECR*, *PAH*, and *CP*. Cytokine signaling pathways enriched at T4 in cattle that were eventually affected by BRD included other interleukin signaling (*SPTA1*, *PRTN3*) and regulation of cytokine production (*ERMAP*).

### 3.5. TIME × VAX Interaction: Temporal Dynamics of Vaccination

The interaction between TIME and VAX resulted in 3001 DEGs, representing one of the largest sources of variation in the dataset, indicating that the vaccination response changed across timepoints (Supplemental File S6). Key enriched pathways included cytokine signaling in the immune system, signaling by interleukins, interferon-gamma signaling and interferon signaling, signaling by B-cell receptors, antigen processing and presentation, and antigen processing cross-presentation. Significant GO terms included MHC class II protein complex (GO:0042613), antigen binding (GO:0003823), and immune response (GO:0006955).

### 3.6. BRD × VAX Interaction: Vaccination Effect on Future Disease Signatures

The interaction between BRD and VAX resulted in a relatively small number of DEGs (*n* = 306 DEGs) and limited pathway enrichment (Supplemental File S6), where only interferon-γ signaling and interferon signaling reached significance.

## 4. Discussion

### 4.1. Time Influences Global Gene Expression Patterns in Blood

The findings reported here are consistent with the development of both the innate and adaptive immune systems over time. The identification of 5364 TIME DEGs by both glmmSeq and at least one pairwise comparison, the largest source of differential expression in the dataset, underscores profound immune system remodeling during the first seven months of life, encompassing the transition from passive colostral immunity to competent adaptive responses [32,33]. The enrichment of innate immune pathways like neutrophil degranulation, adaptive immunity, and cytokine signaling aligns with the age-dependent expansion of immune cell populations in neonatal calves [32]. The upregulation of these pathways from T1 to T3, followed by the downregulation to T4, suggests an elevation in immune system activation at approximately 183 days of age, followed by the resolution of inflammatory signaling by weaning at 230 days.

SPMs orchestrate and execute inflammatory resolution, and their downregulation from T3 to T4 suggests that active resolution rather than simple immune response deactivation had occurred [34,35,36,37,38]. Whereas these SPM dynamics track developmental immune maturation across the preweaning period, our prior analysis of this cohort showed that marked SPM-associated downregulation occurred specifically at backgrounding entry in the NOVAX AUCTION calves, implicating marketing and transport stress rather than preweaning development [17]. Chronic inflammation can be further exacerbated when resolution pathways become defective [36]; this may increase BRD susceptibility during weaning. The elevation in immune-related gene expression at T3 occurred prior to attenuated viral booster administration and surgical castration, indicating that this elevation reflects the calves’ immune state at 183 days of age, rather than a response to the same-day management tactics, while the subsequent T4 downregulation reflects homeostatic rebalancing before weaning stress [33,39]. However, it is important to consider that all animals were restrained and processed similarly, so the T3 signal cannot be only attributed to vaccination or to the stress of handling individually.

### 4.2. Influences of Vaccination on Gene Expression

Pairwise testing with edgeR identified a greater number of DEGs at individual points, demonstrating the magnitude of gene expression changes between timepoints, whereas the glmmSeq framework is a trajectory-based approach that identified genes over the entire sampling period, which in turn returned fewer significant genes. The unique clustering pattern at T3 suggests a transient long-term vaccine-induced transcriptional response approximately 76 days following initial vaccination [39,40].

Revaccination is expected to induce an anamnestic immune response, when memory B and T cells formed at initial vaccination respond more rapidly at higher magnitudes and with greater antibody affinity than at the first encounter [39]. Given this understanding, we expected more DEGs between T3 and T4. Although we may not have sampled at the timepoint when DEGs were most differentially expressed following revaccination at T3, VAX samples were most separated from NOVAX samples, suggesting that vaccination has a more prolonged effect even before revaccination. The enrichment of the antigen processing, heat shock response, and phagocytosis pathways reflects active immune engagement, ongoing antigen presentation, and cellular machinery remodeling persisting for longer than the expected acute response window. In human systems vaccinology, it has been demonstrated that blood transcriptomics captures vaccine-induced molecular patterns that are predictive of downstream antibody responses across diverse vaccine platforms [9]. Whether DEGs measured at industry-relevant timepoints could be used to infer respiratory vaccination status in cattle for which vaccination records are unavailable represents further work that is encouraged by these findings. No classification framework was developed or evaluated here, and no measure of classification accuracy was generated; this possibility is therefore a hypothesis for future work.

Across all timepoints, we expected the enrichment of classical antiviral signaling pathways, and the absence of a detectable signal is notable. The absence of a detectable signal related to antiviral signaling during the preweaning period is consistent with our prior analysis of this cohort, in which type I interferon-stimulated genes were not induced until backgrounding entry and then primarily occurred in NOVAX AUCTION calves, suggesting that viral exposure occurred during marketing rather than preweaning [17]. The clostridial bacterin–toxoid administered to all calves at T1 and T3 was not expected to drive DEGs related to type I interferon signaling because it was applied uniformly to all groups; thus, clostridial vaccination does not explain the absence of antiviral enrichment in the NOVAX vs. VAX comparison. As hallmarks of antiviral immunity that promote interferon-stimulated gene expression, type I interferons bridge innate and adaptive immune activation and serve as endogenous vaccine adjuvants that enhance humoral and cellular immune responses [41,42]. Their absence is notable, and there are at least two mechanistic explanations that may account for this observation. First, acute type I interferon responses are thought to peak within hours to days of viral exposure and then are downregulated quickly, so the 7-day interval between T1 and T2 may have resulted in missing the acute IFN window [9]. Second, vaccine strains of attenuated vaccines possess reduced virulence and may not trigger an immune response that is as robust as that to a wild-type infection, resulting in a functional yet dampened response. Despite failing to detect a difference in type I IFN signatures in the VAX main effect, the TIME × VAX enriched pathways related to the IFN-γ and interferon signaling pathways suggest that the interferon response to vaccination is highly time-dependent rather than not affected altogether [11,43]. However, antigen presentation pathways were persistently enriched, suggesting prolonged antigen processing activity following modified live virus vaccination and the development of a robust immune response [43].

### 4.3. Preclinical BRD-Associated Gene Expression Patterns

The identification of BRD-associated gene expression at T4, before transport and entry into the backgrounding facility, suggests that host gene expression at weaning could be indicative of subsequent BRD susceptibility during the high-risk backgrounding period [39,40,44,45,46]. The 129 BRD-associated DEGs, strongest at T4, provide evidence that transcriptomic susceptibility signatures precede clinical disease [13,14]. In this study, the last intervention point before backgrounding was at T4, with samples taken before cattle were weaned [39]. The significant pathways at T4 included hemopoiesis with the enrichment of oxygen/CO_2_ exchange and hemoglobin processes. Key genes included *RHAG* (Rh-associated glycoprotein), *AHSP* (alpha hemoglobin stabilizing protein), and *SPTA1* (spectrin alpha 1), suggesting altered erythroid and oxygen transport activity (Supplemental File S8). One hypothesis is that reduced oxygen-carrying capacity impairs oxygen-dependent immune functions such as neutrophil respiratory burst and T-cell proliferation; alternatively, these findings could reflect early subclinical pulmonary abnormalities affecting gas exchange [47]. Neither hemoglobin concentrations, erythrocyte indices, oxygen transport physiology, nor pulmonary function were measured in this study, so both explanations are offered as hypotheses for future work. In BRD cattle, complementary metabolic pathway enrichment, fatty acid metabolism (*MECR*), phenylalanine metabolism (PAH), and oxidation reduction suggest preexisting metabolic inefficiency, suggesting the reduced ability of the immune system to respond. Cytokine dysregulation at T4, similarly to interleukin signaling driven by *PRTN3* and *ERMAP*, may possibly indicate that immune gene expression patterns accumulate before pathogen exposure [48].

Following the confirmation and formal validation of these associations, future practical applications of these findings could include precision interventions where cattle could receive enhanced vaccination, metaphylaxis, nutritional support, or segregation into lower-stress cohorts to decrease BRD following weaning and transport [47,49,50,51]. Current BRD diagnosis is reliant on the visual assessment of cattle in pens, which has demonstrated poor sensitivity but high specificity, highlighting the need for more accurate clinical identification tools and techniques [52,53]. However, the future field deployment of gene expression would need to be further evaluated to ensure practicality, validity in larger populations, and efficiency.

### 4.4. Time Modulates the Effect of Vaccination on Gene Expression

The enrichment of the MHC class II and antigen presentation pathways may suggest that antigen processing capacity or activity changed substantially from birth through weaning in VAX vs. NOVAX cattle, possibly helping to explain the unique clustering of T3 VAX samples. The TIME × VAX interaction demonstrates that T3, where vaccination status effects are most pronounced, is possibly driven by ongoing immune maturation and a sustained response to primary vaccination [32,40].

The TIME × VAX interaction (3001 DEGs) demonstrates that vaccination responses are profoundly modulated by the development state, with increased differentiation at T3. The unique clustering of T3 VAX samples demonstrates a transient vaccine-specific transcriptional state 76 days post-primary vaccination [39]. The T3 VAX signature was dominated by heat shock response pathways driven by genes such as *HSPA4*, a molecular chaperone that is critical for antigen processing and MHC class I presentation. This signature likely reflects sustained antigen processing activity and activity associated with the development of memory from the primary vaccination, together with age-related immune maturation at approximately six months, rather than acute viral replication, since the animals were not diagnosed with BRD during these sampling timepoints [41,42]. In contrast, the TIME × VAX interaction pathways were enriched for IFN-γ and interferon signaling, indicating that interferon responses in vaccinated calves were time-dependent rather than sustained [41,42]. Persistent antigen presentation pathway enrichment, particularly that of MHC class II, suggests prolonged antigen availability, supporting durable memory formation [9].

### 4.5. Vaccination Effects on Future BRD Expressional Signatures

The limited number of enriched pathways and the small number of genes driving these enrichments suggest that preweaning vaccination did not correlate strongly with distinct disease phenotypes that could be detected through peripheral blood transcriptomes at T1–T4. The limited BRD × VAX interaction with minimal DEGs and sparse enrichment contrasts the robust TIME × VAX interaction, indicating that vaccination was associated with substantial differences in the developmental responses of the immune system but was not associated with subsequent BRD susceptibility identifiable under the conditions of this study. Three mechanisms may explain this. The first is possible compartmentalization, where vaccine protection is focused through the local respiratory mucosal immunity that is not reflected in the peripheral blood [54]. Second, the acute vaccination-associated responses may not have been fully captured, as the early post-vaccination window was not sampled directly in our sampling timepoints [9]. Third, intrinsic host factors like genetics, nutrition, stress resilience, and maternal effects on BRD-associated gene expression pathways may influence disease susceptibility despite vaccination history [55]. The similar T4 pathway enrichment in cattle later diagnosed with BRD, regardless of vaccination, suggests that host response factors, rather than vaccine-modified immune status, are quantifiable and targetable. Practically, this is a positive indication for the future development of biomarkers using blood transcriptomic signatures for BRD risk stratification that apply equally across vaccinated and unvaccinated populations, enhancing the translation potential by not requiring vaccination status as a covariate to render these signatures useful for predicting risk. Importantly, this does not imply that vaccination is ineffective, as clinical trials demonstrate reduced BRD incidence in vaccinated cattle; rather, it suggests that preweaning attenuated viral vaccine-associated protection mechanisms are not captured by peripheral blood transcriptomics at the predisease timepoints evaluated in this study [7].

### 4.6. Principal Findings and Limitations

This longitudinal transcriptomics study revealed four principal findings. First, regardless of vaccination or future BRD diagnosis, TIME strongly influences gene expression related to the immune system, reflecting developmental immune maturation from birth to weaning. Second, attenuated viral vaccination was associated with immune-related gene expression, with the strongest measurable signal at approximately two months after the first vaccination; this was characterized by the heat shock response and antigen presentation pathways. Third, preclinical transcriptomic differences at weaning (T4) may be associated with subsequent BRD susceptibility during backgrounding independently of vaccination status, identifying candidate genes for future biomarker development. Fourth, the BRD × VAX interaction produced few DEGs and minimal pathway enrichment, indicating that vaccination status did not modify BRD-associated gene expression in this cohort; a transcriptome risk signature may not need to be dependent on vaccination history.

Limitations of this study include the temporal resolution of our sampling. Although the four timepoints were selected to align with industry-relevant management events (initial vaccination, revaccination and castration, and weaning) and capture critical immunological development, this design meant that sampling did not necessarily coincide with the acute and early immune responses of greatest interest or necessarily capture the full dynamic range of the immune response. Calves received their initial vaccination at a median age of 107 days, an age at which maternally derived antibodies may still be present in some individuals, which is known to interfere with the response to parenteral respiratory vaccines [56]. Variation in maternally derived antibodies is an unmeasured source of heterogeneity in the vaccine-associated transcriptional response described here and may have contributed to the small number of VAX main-effect DEGs and to the delayed timing of the strongest vaccine-associated signal at T3. Additionally, while we have demonstrated that whole-blood transcriptomes can be associated with clinical disease status and focused on a convenient sample, our work may not reflect all aspects of local respiratory immunity within the lung tissue [8,9,10]. Moreover, generalizability is limited due to the fact the work was conducted in one group of cattle with a single study site/herd, and, while the study was designed to represent common production settings and common management practices in North America, the setting was still of notably lower risk compared to other higher-risk production methods [4]. It is possible that different immune development dynamics, gene expression signatures, and health outcomes could be seen in cattle that are managed differently. Future work should incorporate sampling timepoints that are closer together and more frequent between T2 and T3; transcriptomics of the respiratory tract; multiomics integration and multisite validation; the parallel measurement of antibody titers and antigen-specific cellular responses; and formal predictive modeling with cross-validation and independent sensitivity, specificity, and predictive analyses.

## 5. Conclusions

This study provides one of the first comprehensive longitudinal blood transcriptome characterizations in beef cattle from birth through weaning, suggesting that temporal immune maturation is the dominant source of variation in gene expression. Attenuated viral vaccination was associated with age-dependent signatures that could enable vaccination history prediction. In the future, preclinical BRD biomarkers at weaning may be used to predict subsequent disease independently of vaccination status. These findings establish a hypothesis for future work that could lead to precision livestock health management that includes transcriptomic risk profiling, genomic selection, and optimized vaccination options to enhance animal welfare and production sustainability.

## Figures and Tables

**Table 1:** Representative differentially expressed genes (DEGs) for each major comparison. Main-effect genes were identified by both glmmSeq and pairwise quasi-likelihood F-testing (QLF): values shown are the QLF log-fold change and Benjamini–Hochberg-adjusted *p*-value (FDR) for the timepoint indicated. Interaction genes were identified by glmmSeq only and have no corresponding pairwise estimate.

Comparison	Gene	log2FC	FDR	Biological Relevance/Enriched Pathway
TIME (T1 vs. T3)	*HSPA6*	−3.186	1.54 × 10^−8^	Heat shock protein family A member 6; cellular stress response
	*HSPA1A*	−1.782	2.12 × 10^−8^	Heat shock protein family A member 1A; cellular stress response
	*ALOX15*	−0.589	1.57 × 10^−2^	Specialized pro-resolving mediator biosynthesis; D-series resolvin and protectin synthesis
	*ALOX5*	−0.584	1.65 × 10^−2^	Specialized pro-resolving mediator biosynthesis; leukotriene and eoxin synthesis; arachidonic acid metabolism
VAX vs. NOVAX (T3)	*ZFAND2A*	−2.848	2.51 × 10^−32^	Zinc finger AN1-type 2A; heat shock responsive
	*HSPA1A*	−3.642	1.78 × 10^−28^	Heat shock protein family A member 1A
	*DNAJA4*	−1.725	3.80 × 10^−25^	DNA heat shock protein family member A4; co-chaperone activity
	*HSPA4*	−0.908	2.97 × 10^−21^	HSF1-mediated heat shock response; chaperone activity supporting antigen processing and presentation
BRD vs. NO BRD (T4)	*PRTN3*	−4.280	2.02 × 10^−5^	Proteinase 3; interleukin signaling
	*RHAG*	−3.740	9.98 × 10^−5^	Rh-associated glycoprotein; erythroid function, oxygen and carbon dioxide exchange
	*SPTA1*	−3.350	1.27 × 10^−4^	Spectrin alpha 1; erythrocyte membrane structure; interleukin signaling
	*PAH*	−2.325	3.04 × 10^−2^	Phenylalanine hydroxylase; phenylalanine metabolism
	*AHSP*	−1.611	3.60 × 10^−5^	Alpha hemoglobin stabilizing protein; hemoglobin stability
	*ERMAP*	−1.128	3.70 × 10^−4^	Erythroblast membrane-associated protein; regulation of cytokine production
	*CP*	−0.412	4.83 × 10^−2^	Ceruloplasmin; oxidation–reduction processes
	*MECR*	−0.198	7.20 × 10^−4^	Mitochondrial trans-2-enoyl-CoA reductase; fatty acid metabolism
TIME × VAX	*HSPA1A*	n/a	n/a	Identified by glmmSeq only; heat shock response
	*HSPA4*	n/a	n/a	Identified by glmmSeq only; MHC class II protein complex (GO:0042613), antigen binding (GO:0003823)
BRD × VAX	*ACAN*	n/a	n/a	Identified by glmmSeq only; extracellular matrix organization and degradation
	*TNC*	n/a	n/a	Identified by glmmSeq only; extracellular matrix organization; IGF transport regulation
	*LAMB1*	n/a	n/a	Identified by glmmSeq only; extracellular matrix organization; IGF transport regulation

## Data Availability

The original data presented in the study are deposited in the National Center for Biotechnology Information Gene Expression Omnibus (NCBI-GEO), accession number GSE248477. Previously generated data utilized by this study are found in the NCBI-GEO, under accession number GSE205004. The original contributions presented in this study are included in the article/Supplementary Material. Further inquiries can be directed to the corresponding authors.

## Supplementary Materials

The following supporting information can be downloaded at https://www.mdpi.com/article/10.3390/vaccines14080694/s1. File S1: Blood Sampling Timeline; File S2: Animal Metadata for Beef Calves Enrolled; File S3: Differentially Expressed Genes Identified Across Timepoint Comparisons; File S4: Differentially Expressed Genes Identified Between BRD and NOBRD Beef Calves Across All Four Timepoints; File S5: Differentially Expressed Genes Identified Between VAX and NOVAX Beef Calves Across All Four Timepoints; File S6: Differentially Expressed Genes and Functional Enrichments Identified by the Interaction between TIME and VAX and BRD and VAX in Beef Calves; File S7: Functional Enrichment Analysis of Differentially Expressed Genes Identified by TIME in Beef Calves; File S8: Functional Enrichment Analysis of Differentially Expressed Genes at Between BRD and NOBRD Beef Calves Across All Timepoints; File S9: Functional Enrichment Analysis of Differentially Expressed Genes at Between VAX and NOVAX Beef Calves Across All Timepoints.
