# Supplementary material for: Impact of Preweaning Vaccination on Host Gene Expression Patterns Linked to Future Bovine Respiratory Disease Development in Beef Calves
Source: Vaccines (Basel). 2026 Aug 12;14(8):694. doi: 10.3390/vaccines14080694 (PMC13517652; doi:10.3390/vaccines14080694)

Supplemental File S1:

A. Supplemental File S1: Blood Sampling Timeline. Split-plot randomized controlled trial in 81 beef bull calves. Blood was collected into Tempus Blood RNA tubes at four timepoints (T1-T4). BRD status was assigned during the 45-day backgrounding phase that followed.

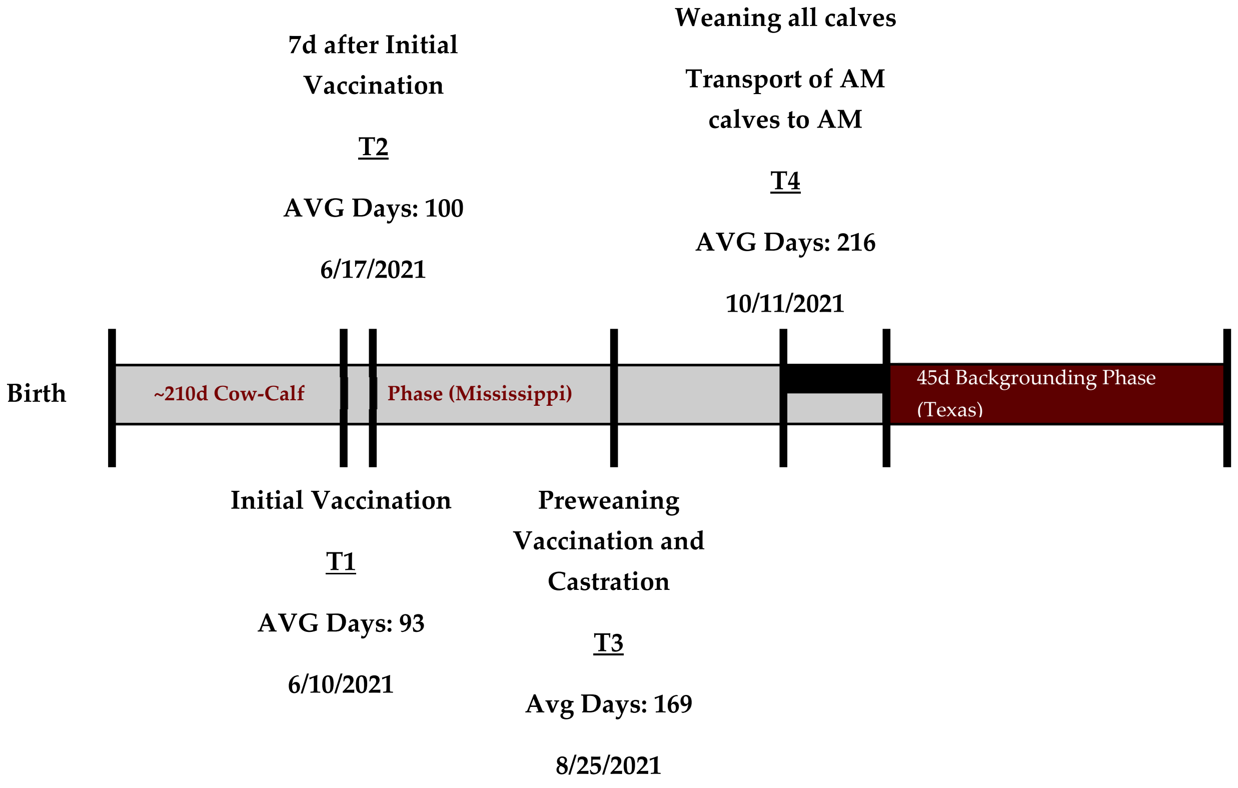

Supplement: Supplementary file 1 [file vaccines-14-00694-s001.zip › S1-Supplemental File S1.pdf]
